# Temperature thresholds of physically dormant seeds and plant functional response to fire: variation among species and relative impact of climate change

**DOI:** 10.1002/ece3.973

**Published:** 2014-02-12

**Authors:** Mark K J Ooi, Andrew J Denham, Victor M Santana, Tony D Auld

**Affiliations:** 1 Institute for Conservation Biology & Environmental Management, School of Biological Sciences, University of WollongongWollongong, New South Wales, 2522, Australia; 2 Office of Environment & HeritageP.O. Box 1967, Hurstville, New South Wales, 2220, Australia; 3 School of Environmental Sciences, University of LiverpoolLiverpool, L69 3GP, U.K; 4 Fundación de la Generalitat Valenciana Centro de Estudios Ambientales del Mediterráneo (CEAM), Parque Tecnológico Paterna. C/ Charles Darwin14, E-46980 Paterna, Valencia, Spain

**Keywords:** Bet-hedging, Fabaceae, fire adaptation, fire management, germination, hard-seeded, heat shock, physical dormancy, seed bank, seedling emergence

## Abstract

Variation in dormancy thresholds among species is rarely studied but may provide a basis to better understand the mechanisms controlling population persistence. Incorporating dormancy-breaking temperature thresholds into existing trait frameworks could improve predictions regarding seed bank persistence, and subsequently species resilience in response to fire, climate change and anthropogenic management. A key ecological strategy for many species from fire-prone ecosystems is the possession of a long-lived seed bank, ensuring recovery after fire. Physical dormancy is dominant in these ecosystems and maintaining this dormancy is directly linked to seed bank persistence. We identified a suite of seed-related factors relevant to maintaining populations in fire-prone regions for 14 co-occurring physically dormant species. We measured variation in initial levels of dormancy and then applied experimental heating treatments, based on current seasonal temperatures and those occurring during fires, to seeds of all study species. Additionally, higher seasonal temperature treatments were applied to assess response of seeds to temperatures projected under future climate scenarios. Levels of germination response and mortality were determined to assess how tightly germination response was bound to either fire or seasonal cues. Six species were found to have dormancy cues bound to temperatures that only occur during fires (80°C and above) and were grouped as having obligate pyrogenic dormancy release. The remaining species, classified as having facultative pyrogenic dormancy, had lower temperature dormancy thresholds and committed at least 30% of seeds to germinate after summer-temperature treatments. Evidence from this study supports including dormancy-breaking temperature thresholds as an attribute for identifying functional types. High temperature thresholds for breaking dormancy, found in our obligate pyrogenic group, appear to be a fire-adapted trait, while we predict that species in the facultative group are most at risk to increased seed bank decay resulting from elevated soil temperatures under projected climate change.

## Introduction

The interaction between critical life-history stages and the environment can determine the relative success, distribution, and level of persistence of species within an ecosystem over time (Harper 1977; Noble and Slatyer 1980; Woodward 1987). For plant communities from fire-prone regions, species can persist using two broad functional approaches: resprouting and recruitment from seed (Pausas et al. 2004). For the latter group in particular, the establishment phase after disturbance is important due to the small window of opportunity for successful recruitment to take place and the need to replace individuals killed by fire (Whelan 1995). Thus, a critical ecological strategy for many species from such ecosystems is the possession of a long-lived seed bank. Seed banks permit long-term persistence for a species when environmental or biotic conditions are unfavorable for other life stages (Grime 1989; Fenner and Thompson 2005), as well as a bet-hedging capacity (Philippi 1993; Ooi et al. 2009). Combined with germination that is triggered by fire cues, this strategy ensures that seedlings emerge during the postfire period into an environment with low levels of competition and high availability of resources (Purdie 1977; Auld et al. 2000). For many species, unsuitable conditions for seedling survival and growth at other times mean that recruitment between fires is rare (Keeley 1987; Meney et al. 1994; Herranz et al. 1999; Ooi et al. 2007). Loss of dormancy and promotion of germination during this interfire period results in a net loss to the seed bank (Ooi 2012).

The majority of species in fire-prone plant communities have soil-stored seed banks (Parker and Kelly 1989; Auld et al. 2000; Holmes and Newton 2004). Species with canopy-stored seed banks usually have nondormant seeds, and the timing of germination is controlled primarily by the timing of seed release after fire (Baskin and Baskin 1998). For soil-stored seeds, physical dormancy, also known as “hard-seededness,” is a dominant mechanism controlling seed bank dynamics (Baskin and Baskin 1998; Ferrandis et al. 1999; Keeley and Fotheringham 2000; Merritt et al. 2007; Ooi 2007). Seeds with physical dormancy have an impermeable coat that prevents water uptake which, once broken, cannot be reversed (Baskin et al. 2000). As such, measuring the proportion of dormancy loss in physically dormant seed lots is a good proxy for estimating seed bank decline. In fire-prone regions, physical dormancy is often overcome by a short duration heat shock related to the passage of fire, resulting in a flush of germination (Auld and O'Connell 1991; Keeley 1991). Several studies have shown that temperatures related to summer conditions or gaps in the canopy can also break physical dormancy (Auld and Bradstock 1996; Baeza and Roy 2008; Santana et al. 2010, 2013; Ooi et al. 2012). In a novel study by Moreira and Pausas (2012), high-temperature fire cues and a seasonal heat treatment were tested for six species from Europe. They concluded that temperature thresholds necessary to break dormancy were better explained as a response to fire, because heat shock maximized germination. However, response to both low-and high-temperature treatments in other studies (e.g., Auld and O'Connell 1991; Herranz et al. 1999; Scuderi et al. 2010) suggests that variation between species is very likely. Additionally, measuring only maximal germination may not be a robust assessment because many species respond to seasonal temperatures by committing only a small proportion of seeds to germinate (e.g., Auld 1995; Ooi et al. 2009). Determining the extent of variation to a range of seasonal and fire temperatures could provide key information for understanding how seed banks function and how species coexist.

Grouping species by functional traits allows predictions for large numbers of species based on the knowledge of relatively few. For example, in fire-prone ecosystems, two functional traits, resprouting capacity and type of seed bank (canopy vs. soil), are used as a means to assess relative resilience of populations in the face of different fire regimes (Pausas et al. 2004; Keith et al. 2007). However, more detailed seed traits have rarely been incorporated into identifying functional groups or understanding their response (e.g., Paula and Pausas 2008). Assessing the variation in threshold temperatures that leads to dormancy-breaking and mortality across co-occurring species can identify those species that are more tightly bound to fire and offer insights into the relative roles that both fire and seasonal cues have played in shaping patterns in physical dormancy (Moreira and Pausas 2012; Santana et al. 2013). It can also help us to identify the mechanisms behind different responses of species that are currently classified within the same functional group (i.e., soil seed bank species) and inform the structure of future plant functional types for assessing the response of groups of species to fire.

Climate projections indicate that many environmental factors, including mean air temperatures, soil conditions, and events such as drought and heat waves, will change in the future (IPCC 2007; Alexander and Arblaster 2009; Royer et al. 2011). Climate has a strong influence on seed dormancy and germination (Walck et al. 2011), and a small increase in mean air temperatures can increase associated soil temperatures to much higher levels (Gutterman and Gozlan 1998; Ooi et al. 2009, 2012). The potential impact on soil seed bank persistence, and subsequent population persistence, particularly for physically dormant species, will depend on the resistance of seed banks to depletion by increased germination and mortality due to higher soil temperatures (Ooi 2012). Loss of physical dormancy is directly related to temperature, with an increasing proportion of a seed lot losing dormancy as temperature increases (Martin et al. 1975; Auld and O'Connell 1991). The direct relationship between temperature and seed bank persistence makes physical dormancy an ideal candidate for studying the impact of projected temperature increase.

Incorporating information on threshold temperatures controlling physical dormancy into existing trait frameworks would allow improved predictions of seed bank persistence and species resilience in response to climate change-related temperature increases. Species with higher dormancy-breaking temperature thresholds (more closely bound to fire temperatures) would be less susceptible to increasing ambient temperatures compared to co-occurring lower temperature threshold species, because seeds would remain dormant in the seed bank during the interfire period. In a recent study, seed bank longevity for two species was found to be compromised by projected summer temperature increases, due to the low threshold temperatures required to break dormancy (Ooi et al. 2012). Auld and Denham (2006) also found a relationship between the magnitude of the residual seed bank after fire and depth and suggested that temperature was the main determinant of seed bank dynamics of physically dormant seeds because heating of the soil profile decreases with depth.

In addition to supporting population recovery after disturbance, by producing germination, seed banks also stabilize population dynamics by spreading risk (Grime 1989). Seed traits therefore need to fulfill dual roles. Firstly, they need to enable the persistence of the seed bank, both during the interfire period and directly after fires. Secondly, they must ensure that emergence is timed to a period conducive to successful recruitment. In our study, we build on previous work investigating summer versus fire germination response (e.g., Moreira and Pausas 2012; Ooi et al. 2012; Santana et al. 2013) by widening the focus from maximal germination response and identifying a set of key seed traits and attributes required for maintaining population persistence in a fire-prone region. We addressed the following questions:

1. Which seed traits or attributes and their related seed bank responses are beneficial for population persistence in fire-driven ecosystems?
2. Do responses of seeds to both fire-related heat shock and summer soil temperatures vary between physically dormant species from the same climatic region and can they be grouped on this basis?
3. If so, what are the relative consequences of climate change-related increase in soil temperatures on seed bank persistence for species within different dormancy threshold groups?

In addition to our main aims, we also discuss whether there is any evidence supporting fire or season as an evolutionary force shaping physical dormancy in fire-prone regions. We conducted this study using 14 obligate-seeding species from south-eastern Australia. Selective pressure of fire would be greatest on seed traits controlling seed bank dynamics in obligate seeders, because they are killed by fire (Paula and Pausas 2008). Compared to resprouting species, this places a much greater dependence on postfire recruitment from the seed bank.

## Methods

### Conceptual framework for identifying fire-and season-related seed traits

In fire-prone regions, high levels of recruitment after fire are necessary for species persistence. The seed bank therefore has to maintain a pool of seeds between fires and deliver those seeds as seedlings at the right time. Selective pressure on physical dormancy would impact upon seed traits that influence both of these roles and, as outlined by Moreira and Pausas (2012), may result from fire or from summer temperatures, where dormancy of the seed bank is lost gradually over each summer season and/or in gaps (e.g., Van Assche et al. 2003; Van Assche and Vandelook 2006; Ooi et al. 2009). To identify the key differences between fire-adapted and gap-detecting species, a conceptual framework was developed, outlining the potential functional mechanisms of seed traits and attributes for each group (Table 1). Seed traits were chosen in relation to three key elements: maintenance of a persistent soil seed bank; germination in response to fire; and risk-spreading or bet-hedging capacity. The key attribute that was hypothesized to distinguish fire-adapted from gap-detecting species was the fire-related heating temperature required to break dormancy. We then looked for the likely strength of other traits or attributes in fire-adapted species (denoted as F in Table 1) versus those responding to seasonal temperatures in gaps (G). For example, we expected no difference in initial dormancy (Trait 1i, Table 1) because F and G species require postdisturbance conditions for successful recruitment and would not benefit from having seeds germinating immediately after dispersal. Conversely, differences in temperature-related dormancy thresholds were expected in response to seasonal heat treatments (Trait 1ii(b), Table 1), because G species need to germinate into gaps, whereas F species need to maintain seed bank persistence until fire.

**Table 1:** Conceptual framework identifying seed bank behavior relevant for population persistence of strongly fire-adapted (F) or gap-responding (G) species in fire-prone environments.

Seed bank behavior promoting persistence in fire-prone habitats	Measurable seed trait/attribute	Relative predicted strength of pattern between fire-adapted (F) and gap-responding (G) species	Rationale
(1) Maintain large seed bank between fires	(i) Highly dormant at dispersal	F: high initial dormancy levels	F and G: Recruitment ideally restricted to postfire environment or gaps, so no benefit would be gained by an initial large nondormant fraction
		G: high initial dormancy levels	
	(ii) Maintenance of dormancy over time by:	F: Low viability loss per summer	F and G: High levels of seed survival needed to maintain persistence soil seed bank between disturbances
	(a) Low levels of mortality	G: Low viability loss per summer	
	(b) Dormancy-breaking threshold temperatures greater than those produced by summer heat in gaps	F: Very low dormancy loss after treatment at summer gap temperatures	F: Dormancy loss and subsequent germination into unsuitable conditions between fires would result in seed bank decay and reduce the number of seeds available for postfire regeneration
		G: Significant dormancy loss after treatment at summer gap temperatures	G: Dormancy broken by seasonal temperature cues would promote germination into gaps
(2) Produce postfire germination flush	(i) Dormancy thresholds related to fire-produced temperatures	F: Significant germination response restricted to temperatures above fire-related threshold (80°C)	F: Dormancy-breaking cues result in a flush of postfire germination in response to heat related to fire, but remain dormant in response to summer soil temperatures
		G: Significant germination at temperatures above gap-related thresholds (40-60°C)	G: Species adapted to respond to summer soil temperatures produced in gaps would have correspondingly lower fire-related thresholds for breaking dormancy
	(ii) Can survive fire	F: Little loss of viability at short duration hot fire temperature	F: Fire-adapted seeds would have to withstand temperature typically related to fire to ensure a flush of germination
		G: Significant loss of viability at short duration hot fire temperature	G: Temperature from fires likely to exceed viability thresholds for species adapted to lower dormancy-breaking cues and a proportion seeds would suffer mortality
(3) Bet-hedging capacity	(i) Persistence and viability postfire	F: significant proportion of seeds remain dormant after fire temperatures	F: Some seeds maintained in the seed bank postfire would prevent a risky all-or-nothing recruitment strategy. Seeds strongly adapted to fire would employ a risk-spreading mechanism in the case of loss of a postfire cohort
		G: zero or limited proportion of seeds remain dormant after fire temperatures	G: Lower dormancy-breaking threshold temperatures would reduce capacity to bet hedge under high fire-related temperatures

### Study species and seed collection

**Figure 1:**
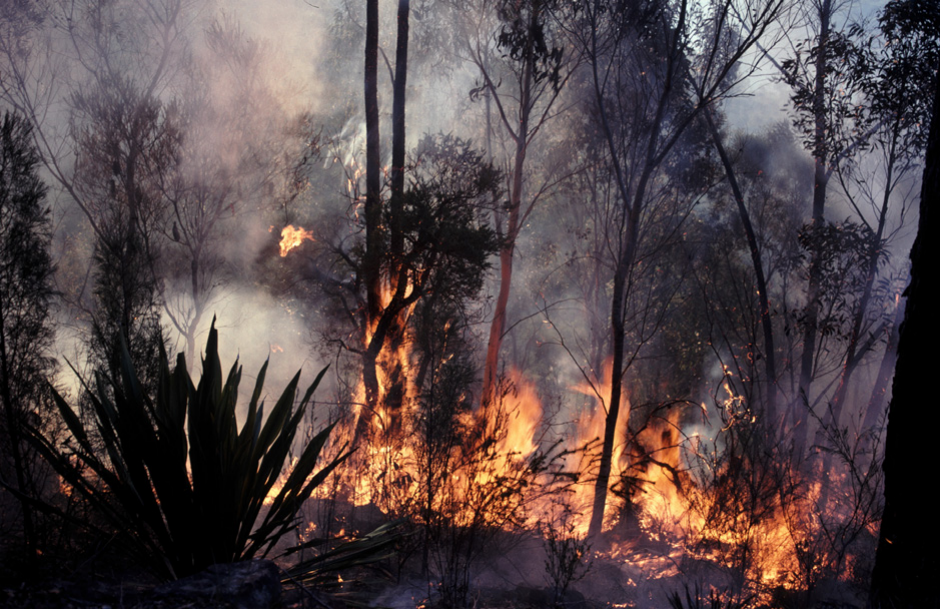
A low-intensity fire burning in Heathcote National Park, in the southern part of the fire-prone Sydney region in south-eastern Australia. Fires are a natural occurrence in these ecosystems.

In fire-prone ecosystems, including South Africa, the USA, the Mediterranean basin, and Australia, there are thousands of species from families known to have physical dormancy. One such ecosystem, where fire is a natural part of ecosystem dynamics, is the Sydney Basin in New South Wales (NSW), south-eastern Australia (Fig. 1), which is a temperate climate region with no dry season. Fire-prone vegetation mainly occurs on nutrient poor sandstone soils and consists of sclerophyllous heath and woodland, with an open canopy of *Eucalyptus, Corymbia,* and *Angophora* species. There is a rich shrub understorey component, dominated by species from the Proteaceae and Myrtaceae, which maintain canopy-stored seed banks with no seed dormancy, and Ericaceae, which maintain soil seed banks via seeds with physiological dormancy mechanisms (Ooi et al. 2006, 2007). The Fabaceae are the most dominant family representing physically dormant species. Over 40% of all shrub species displaying seed dormancy in the Sydney region have physical dormancy (Ooi 2007).The main physically dormant families represented are the Fabaceae, Rhamnaceae, and Sapindaceae. The Fabaceae are the most numerous, with 230 native species across 33 genera in the Sydney region. They represent approximately 10% of the total vascular flora (Auld 1996) and 20% of the shrub component (Ooi 2007). We conducted experiments on 12 common and widespread shrub species, all in the Fabaceae except *Dodonaea triquetra* (Sapindaceae) and *Pomaderris discolor* (Rhamnaceae) (Table 2). We included data for two additional species, *Bossiaea obcordata* and *B. rhombifolia* (Fabaceae), from previous experiments conducted within the same study region (Santana et al. 2010). Nomenclature follows Harden (1990, 1991). All species are obligate seeders, so above-ground plants are killed by fire, and the population depends on regeneration from a long-lived soil-stored seed bank for recovery and persistence. Seeds of each species were collected by hand in late 2009 at Heathcote (34°07′S, 150°58′E) and the adjacent Royal (34°03′S, 151°03′E) National Parks (NP), and Garigal NP (33°34′S, 151°11′E), all within the Sydney Basin. They were stored at room temperature for less than four weeks prior to use in germination experiments. For all species, completely ripe open fruits were removed from at least 30 plants per site. For each species and site, seeds were pooled across individual plants to maximize seed numbers for subsequent germination trials.

**Table 2:** Mean seed weight, initial viability, and time to germination of seeds which have had their dormancy broken for all study species.

Species	Mean seed weight (mg)	Initial mean viability (%)	Time to onset germination (*T*_on_) (Days)	Time to 50% germination (*T*_50_) (Days)
*Acacia terminalis*	28.51 ± 0.84	92.3 ± 4.26	4.71 ± 0.36	8.71 ± 1.48
*Acacia ulicifolia*	9.92 ± 0.27	100.0 ± 0.00	16.8 ± 1.32	29.40 ± 1.50
*Acacia suaveolens*	36.35 ± 0.78	98.9 ± 1.12	8.60 ± 0.97	16.60 ± 2.71
*Acacia linifolia*	29.10 ± 0.95	85.3 ± 7.11	17.30 ± 3.66	25.00 ± 1.26
*Dodonaea triquetra*	3.38 ± 0.14	91.7 ± 1.67	9.50 ± 1.63	14.50 ± 0.50
*Pomaderris discolor*	0.79 ± 0.03	93.1 ± 1.62	10.67 ± 0.70	14.00 ± 1.15
*Dillwynia retorta*	5.63 ± 0.10	93.3 ± 3.45	18.43 ± 1.81	27.86 ± 1.78
*Gompholobium grandiflorum*	3.84 ± 0.34	90.6 ± 3.67	13.56 ± 0.52	20.94 ± 1.57
*Bossiaea rhombifolia*	11.86 ± 0.30	86.7 ± 7.26	6.71 ± 0.18	7.86 ± 0.83
*Gompholobium latifolium*	6.10 ± 0.19	94.2 ± 2.39	11.43 ± 0.95	16.29 ± 1.46
*Dillwynia floribunda*	1.86 ± 0.05	95.3 ± 1.01	11.89 ± 0.73	14.95 ± 0.80
*Bossiaea heterophylla*	21.94 ± 0.48	100.0 ± 0.00	13.50 ± 0.67	15.00 ± 0.00
*Bossiaea obcordata*	2.46 ± 0.07	83.3 ± 4.41	12.00 ± 1.29	15.50 ± 1.44
*Pultenaea stipularis*	9.75 ± 0.32	93.3 ± 1.67	26.56 ± 3.51	42.11 ± 3.35

### Experiment 1: Initial seed viability and dormancy levels

Initial viability and germinability and seed weight were tested for fresh seeds within four weeks of collection for all species. Three replicates of between 15 and 30 seeds were placed into three 9-cm-diameter petri dishes on filter paper moistened with distilled water. Dishes were incubated at 25/18°C on 12 h/12 h light/dark and maximum/minimum temperature cycles. Incubation temperatures were set to mimic germination during the summer, which is the time of seed release and the season during which the majority of natural wild fires occur in this region (McLoughlin 1998). Three incubators were used during germination trials, all set to the same temperature regime, to allow replication at the cabinet level. Germination was scored on emergence of the radicle. At the end of 10 weeks, remaining ungerminated seeds were scarified and germination was followed for a further 4 weeks to assess viability, followed by a final inspection via a cut test (Ooi et al. 2004).

### Experiment 2: Seed viability and germinability in response to summer-related heating

In Experiment 2, prior to germinability trials, dry unimbibed seeds were treated by exposure to current soil temperatures in gaps and elevated soil temperatures resulting from increased air temperatures projected from three climate change models (BCM2.0, CSIRO MK3.5, and MIROC-H). We selected an air temperature increase of 4°C to estimate future mean soil temperatures in summer. A 4°C air temperature increase for summer corresponds to the upper bound of the A1FI scenario range for 2070 and the lower bound range for the A1B scenario for 2100 (IPCC 2007).

This experiment was designed to assess the variation in mortality, dormancy, and germination response between co-occurring species to current temperatures and projected increased mean and heat wave temperatures. To determine these treatment temperatures, data for air and soil temperatures experienced by seeds in summer in gaps under current climate conditions were collected from Royal NP in summer 2009. Temperatures applied representing current mean summer soil conditions in gaps were 40/20°C, and those for future mean conditions were estimated at 47/20°C. These were applied for durations of 7, 14, and 30 days. In addition to representing a mean temperature increase, multiple-day durations of soil temperatures of 47/20°C already occur under the current climate. The 7-day duration of this treatment therefore also represents current heat wave events. Future heat wave conditions in the soil were estimated at 55/20°C. Exceptional heat waves of five days or more duration have been recorded five times in the study region (over 153 years of observations), and a recent record-breaking duration of seven days occurred in 2011 (Ooi et al. 2012). Long rain-free periods, and hence maximum soil warming conditions, are also projected to increase in the future (Mpelasoka et al. 2008).We used summer temperatures in canopy gaps for all treatments because summer is the period of seed release, when seeds in the soil would experience the highest temperatures, and to highlight the influence of gaps versus fire on seed response. We used temperatures reached in the upper part of the soil profile because seeds of all the study species are released from the parent plants onto the soil surface during summer. While some seeds are taken into ant nests and are likely to be lost due to being too deeply buried (Auld 1986), a large proportion have their elaiosomes eaten in situ and remain on the surface, or are ejected back out of the nests and likely to remain uncovered or buried only within the very top of the soil profile for significant periods (Auld 1986; Tozer 1998; Marthews et al. 2008). See Ooi et al. (2012) for a fuller explanation of the global climate models selected and how future soil temperature and treatment temperatures were estimated.

We applied dry heat treatments to seeds prior to incubation for germinability tests. The dry heat treatments were conducted in high-temperature-rated incubators. Daily temperature cycling in incubators was set to 9 h/15 h (max/min), based on hourly soil temperatures, which had climbed to at least 10°C above overnight minimums by 8 am, and by 9 am was generally within 5°C of maximum daily temperatures. By 6 pm, soil temperatures had begun significant declines from the daily maximum. Incubators took approximately 30 min to cool down to minimum temperatures during the changeover. For the full experiment, we applied nine temperature x duration treatments and used three replicate dishes per treatment (a total of 27 petri dishes per species). Each dish contained between 15 and 30 unimbibed seeds. Too few seeds were available for the full experiment for five species. For *Dillwynia floribunda,* all treatments except the 30-day duration at 55/20°C were applied, while for *A. linifolia* and *B. heterophylla,* only the 14-day duration was applied at 55/20°C. For *A. terminalis* and *Dillwynia retorta*, the only treatments applied were the 30-day duration at 40/20°C and 47/20°C.

#### Germinability trials

Dry heat treatment start dates were staggered so that subsequent germinability trials could begin simultaneously. Germinability was assessed using the methods described above for Experiment 1. Each 9-cm petri dish contained between 15 and 30 seeds, depending on species seed viability and availability, placed on filter paper moistened with distilled water. Seeds were checked every 2 to 3 days for the first 4 weeks and then weekly up to 10 weeks. Germination was scored on emergence of the radicle. The mean proportion of seeds that germinated was calculated at the end of the 10-week period, based on initial levels of viability. Mortality was estimated based on the difference between seed viability of the control and post-treatment seeds. Viability was assessed as described for Experiment 1.

### Experiment 3: Seed viability and germinability in response to fire-related heating

Dry heat treatments representing a range of typical temperatures experienced in the top 3 cm of the soil profile during fire, where the majority of seeds emerge from (Auld and O'Connell 1991; Bradstock and Auld 1995), were applied to seeds prior to germinability tests. We used four temperature treatments of 40, 60, 80, and 100°C for 10 min. We also applied 120°C for 1 min to assess seed mortality. For each species, we used three replicate dishes per treatment temperature and each dish contained between 15 and 30 unimbibed seeds. Three ovens were used to replicate heat treatments, with species dishes randomly assigned to a spot in the center section of the middle shelf in each oven. After application of fire-related heat treatments, germinability and viability were assessed as described for Experiment 1. Limited seed numbers were available for *Dillwynia retorta*,*G. grandiflorum, B. rhombifolia,* and *B. obcordata*, so we used data compiled from our previous work within the study region.

### Assigning of groups based on response to dormancy-breaking heat treatments

Species were divided into putative response groups based on their germination after heating. We considered that dormancy loss only after fire treatment above 80°C suggested that fire was a strict requirement for germination, while a loss of dormancy of 20% or more of the seed lot after treatments at either current summer gap or heat wave conditions represented a significant summer gap response. This is in the range of dormancy loss for physically dormant species from habitats where fire does not drive population dynamics (e.g., Auld 1995; Van Assche et al. 2003; Ooi et al. 2009) and which display a gradual seasonal loss of dormancy from the seed bank to take advantage of sporadic rainfall events. For each species, we compared dormancy thresholds related to summer gaps and the corresponding fire-related thresholds to see whether there was a relationship between the two heating methods. We then assessed other seed-related attributes to see whether the assigned groups were robust. This included two measures of bet-hedging capacity, with the proportion of dormant seeds remaining after maximum germination (*G*_max_) and time to the onset of germination (i.e., the first germinant) (*T*_on_) and to 50% germination (*T*_50_) identifying a postfire residual seed bank and a mechanism for temporal spreading of germination, respectively. We also assessed seed mortality after different heating treatments.

### Analyses

The mean proportion of viable seeds germinating, mean viability, *T*_on,_ and *T*_50_ were calculated for all experiments. For each species, we used generalized linear models (GLMs) with a binomial error structure and logit link function to compare final germination proportions across treatment temperatures. When treatment effects were significant, we used the treatment contrasts method to compare means of the levels within factors. For species where the full experimental design of three temperatures at three durations was possible, we also used a GLM with temperature and duration included as factors. The same analyses were applied for seed viability data. Where this was not possible, such as for *A. suaveolens* and *Dodonaea triquetra,* where the proportion germinated for some treatments had zero variance, we used arcsine transformed data and linear models.

To assess the relationship between summer gap-and fire-related temperature thresholds, we compared germination response to both current and future summer treatment temperatures against germination response at each of the fire heat treatments using linear regression. To investigate the differences between our putative groups, we calculated the mean minimum fire-related temperatures required to produce 20% (*G*_20_), 50% (*G*_50_), and maximum germination (*G*_max_) for each group of species and compared the means using the Wilcoxon rank-sum test. We used the same approach assessing *T*_on_ and *T*_50_. All analyses were conducted using the open source software R 2.11.1 (R Development Core Team 2010).

## Results

### Initial seed viability and dormancy

Initial viability was high for all species (>80%, Table 2). Initial dormancy was also very high; only *B. obcordata* and *G. grandiflorum* had dormancy levels below 90% (but above 80%) (Table 3). Both T_on_ and T_50_ ranged widely between species (Table 2), from 4.71 to 26.56 days (*T*_on_) and 7.86 to 42.11 days (*T*_50_). There was no noticeable pattern in time to germination, with variable response recorded between four species within the same genus (*Acacia*; Table 2).

**Table 3:** Categorization of species as having either obligate or facultative pyrogenic dormancy release based on results from this study. Data are response to summer and fire treatments for each species. *G*_max_ is the maximum germination reached across all durations of the treatment specified.

Functional group			Response to summer gap temperatures		Response to fire temperatures				
Seed trait/attribute measured‡:		1i	1ii(b)	1ii(b)	2i	2i	2ii	2ii	3i
Species	Pyrogenic dormancy release class	Initial mean dormancy (%)	*G*_max_ after current summer heat (%)	Germination increase above *G*_max_ after projected summer heat (%)	Temperature required for G_20%_	Temperature required for *G*_max_	% mortality after 10 min at 100°C	% mortality after 1 min at 120°C	% dormant seeds remaining post-*G*_max_
(1) *Acacia rminalis*	Obligate	100.00 ± 0.00	0	0	80	100	8.9	8.9	13.3
(2) *Acacia licifolia*	Obligate	97.77 ± 2.23	6.8	1.3	80	100	7.7	0	31.7
(3) *Acacia suaveolens*	Obligate	91.33 ± 8.67	7.0	15.7	100	100	0	0	45.0
(4) *Acacia linifolia*	Obligate	93.50 ± 3.62	9.5	0.6	100	100	0	8.1	23.5
(5) *Dodonaea triquetra*	Obligate	100.00 ± 0.00	14.0	15.1	100	100	0	0	7.0
(6) *Pomaderris discolor*	Obligate	98.15 ± 1.85	14.8	0	80	100	0	1.6	5.7
(7) *Dillwynia retorta*†	Facultative	97.33 ± 2.77	31.4	48.8	60	60	23.3	86.7	2
(8) *Gompholobium grandiflorum*†	Facultative	81.5 ± 4.03	35.8	30.7	40	80	36.7	100	0
(9) *Bossiaea rhombifolia*†	Facultative	90.00 ± 2.89	37.9*	31.4*	60	80	53.3	100	7.1
(10) *Gompholobium latifolium*	Facultative	95.00 ± 5.00	38.8	32.5	60	80	0	15.5	0
(11) *Dillwynia floribunda*	Facultative	98.27 ± 1.73	38.9	7.4	60	60	7.0	18.6	0
(12) *Bossiaea heterophylla*	Facultative	93.33 ± 3.84	51.9	6.7	40	60	6.7	80.0	0
(13) *Bossiaea obcordata*†	Facultative	83.33 ± 6.01	58.8*	41.7*	80	80	6.8	100	7
(14) *Pultenaea stipularis*	Facultative	96.3 ± 1.85	64.6	9.0	40	60	19.9	18.3	3.3

* Denotes additional seasonal data from Santana et al. (2010).

† Denotes species where additional fire data compiled from Auld (unpublished data) and Auld and O'Connell (1991).

‡ Codes relate to the measurable seed traits and attributes outlined in Table 1.

### Seed viability and germinability in response to summer gap-related heating

**Figure 2:**
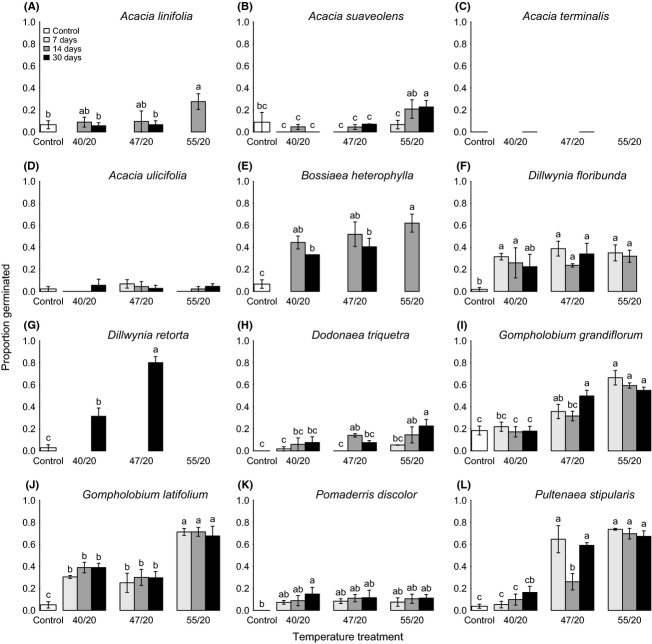
Germination response of the study species (A–L) to heat treatments simulating current and projected future summer temperatures. Bar colors represent durations of heat treatments as per the legend. Different letters above bars indicate significant differences between means. Error bars indicate ±1 SE.

Response to summer gap heat treatments varied between species (Fig. 2). For *A. terminalis*,*A. ulicifolia* and *Pomaderris discolor* (Fig. 2C, D and K) application of summer heat treatments had little or no effect on germination response. For *A. linifolia* and *A. suaveolens,* only germination at 55/20°C (future heat wave) differed significantly from the control (Fig. 2A, B).Germination increased significantly above controls after treatment at 40/20°C or 47/20°C for the remaining species. For species where duration was included as a factor, there were significant main effects for both treatment temperature and duration for *A. suaveolens* (temperature, df = 2, *χ*^2 ^= 25.35, *P *<* *0.001; duration df = 2, *χ*^2 ^= 9.73, *P *<* *0.008) and *Dodonaea triquetra* (temperature, df = 2, *χ*^2 ^= 8.72, *P *<* *0.013; duration df = 2, *χ*^2 ^= 15.60, *P *<* *0.001), with small increases at longer durations at 55/20°C compared to the control. For *Pultenaea stipularis*, there was a significant interaction between temperature and duration (temperature, df = 4, *χ*^2 ^= −14.43, *P *<* *0.006) (Fig. 2L); however, this appeared to be driven mainly by low germination for one replicate at the 47/20°C 14-day treatment. Germination for this species was much higher at the two higher temperature treatments than at the control or 40/20°C. Data from Santana et al. (2010) showed significant increase in germination with summer treatment temperature for *B. rhombifolia* and *B. obcordata*. There was no significant decline of viability as temperature increased for any of the study species.

### Seed viability and germinability in response to fire-related heating

**Figure 3:**
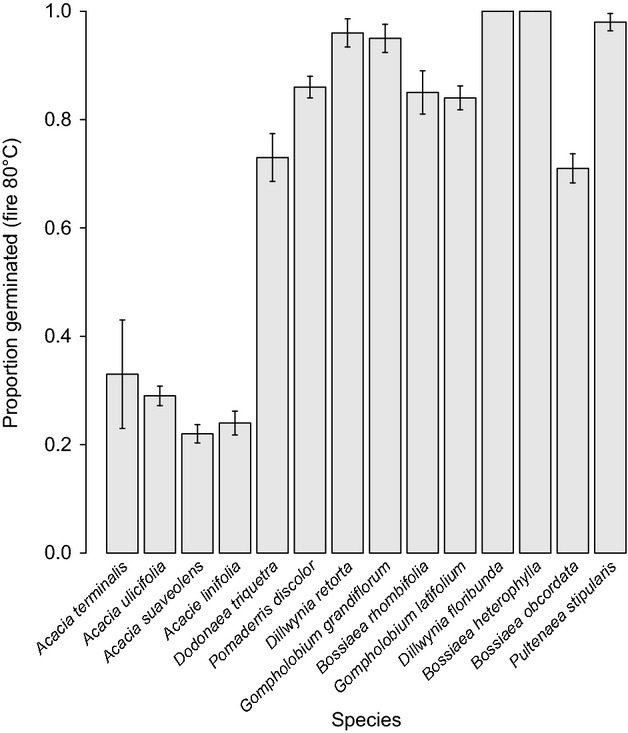
Germination response of the study species after a fire-related heat treatment of 80°C. Error bars indicate ±1 SE.

As expected, fire-related heating broke dormancy for all species but the between-species response to temperature varied considerably. For six species (1–6 in Table 3), the maximum germination (*G*_max_) occurred after treatment at 100°C (Table 3). Germination was less than 5% after the 40°C treatment and less than 25% after 60°C (data not shown). For the remaining species (7–14 in Table 3), *G*_max_ was reached after treatment at temperatures of 60°C or 80°C (Table 3). For the majority of this latter group, the 40°C fire treatment produced germination of between 7% and 46% (with the exceptions being *B. rhombifolia* and *D. floribunda*, which did not germinate at this temperature), and the 60°C treatment produced between 64% and 100% (with the exception of *B. obcordata* which produced 17%). Total germination for each species after treatment at 80°C indicated a low response of < 40% from the four *Acacia* species, between 60% and 80% for the *Dodonaea* and *B. obcordata,* and between 80% and 100% for the remainder (Fig. 3).

At 100°C, a 10-min duration produced no or very low levels of mortality for the *Acacia* species, *Pomaderris discolor* and *Dodonaea triquetra* (1-6 in Table 3). However, mortality at this temperature ranged from approximately 7% and 53% for seven species (Table 3), namely *B. heterophylla*,*B. obcordata, B. rhombifolia, Dillwynia floribunda, D. retorta*,*G. grandiflorum,* and *Pultenaea stipularis*. At 120°C, a 1-min treatment duration had minimal impact on mortality (0-9%) for species 1 to 6 (Table 3), but there was marked mortality between 15% and 100% for species 7-14 (Table 3).

**Figure 4:**
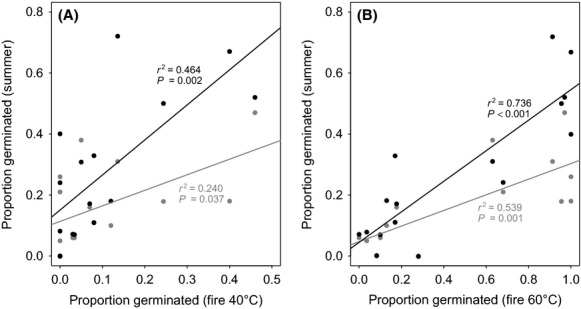
Relationship between germination response at low-temperature fire-related heat treatments of (A) 40°C or (B) 60°C and summer gap heat treatments. Current summer treatment temperature data are presented in gray (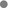) and projected future treatment temperature data in black (•).

There was a positive relationship between germination response to current summer gap temperature treatments and response to low-temperature (40–60°C) fire-related heat treatments (Fig. 4). A positive significant relationship was found between germination after fire heat treatment at 40°C and both current (*r*^2 ^= 0.240) and future summer treatment germination response (*r*^2 ^= 0.464) (Fig. 4A). This was also significant for germination after the 60°C fire heat treatments in relation to current (*r*^2 ^= 0.539) and future germination response (*r*^2 ^= 0.736) (Fig. 4B). At higher fire-related temperatures, this relationship was lost because all species germinated to high levels.

### Comparison of species grouped by dormancy-breaking temperature thresholds

**Figure 5:**
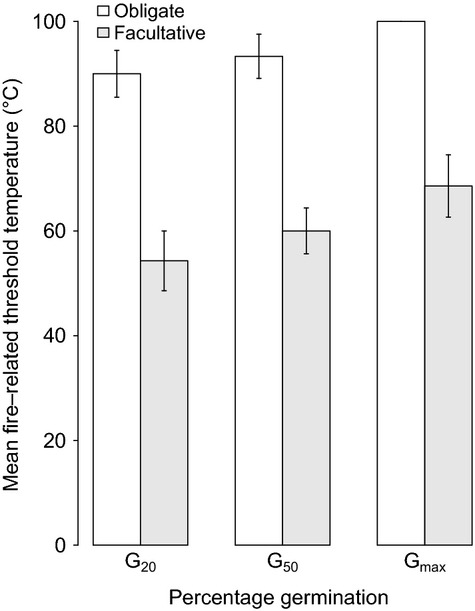
Mean fire-related temperatures required to break 20%, 50%, and maximum germination for the obligate and facultative pyrogenic dormancy release groups of species.

Two clear groupings emerged based on the response to fire-related heating. Summer gap temperature dormancy-breaking treatments and the other seed attributes examined supported the classification. Six of the study species, including the four *Acacia* species, *Dodonaea triquetra* and *Pomaderris discolor*, showed no significant increase in germination response after treatment at current mean summer gap or heat wave temperatures (Table 3). This group also had dormancy-breaking temperature thresholds that could be produced only by fire (≥80°C), displayed high levels of initial dormancy and lower levels of mortality at exposure to high temperatures than the other study species. These six species are therefore considered to have seed traits closely bound to fire and classified as having obligate pyrogenic dormancy release. The mean dormant seed fraction remaining after exposure to the temperature required for *G*_max_ (Table 3) was used as an estimate of the residual seed bank and therefore bet-hedging capability. This indicated that species with obligate pyrogenic dormancy also maintained between 5% and 45% of their seed bank after fire. The remaining species displayed at least 30% germination after current summer gap treatments of 40/20°C or short duration 47/20°C. After treatment at fire-related temperatures and durations, relative to obligate pyrogenic species, they displayed higher losses of viability, lower threshold temperatures for breaking dormancy, and often no residual viable seeds (Table 3). These species are classified as having facultative pyrogenic dormancy release. The mean fire-related temperatures for 20% (*W* = 46.5, *P *<* *0.004), 50% (*W* = 41, *P *<* *0.004), and maximum (*W* = 39, *P *<* *0.005) dormancy release were significantly lower than those with obligate pyrogenic dormancy release (Fig. 5) (Table 3).

**Figure 6:**
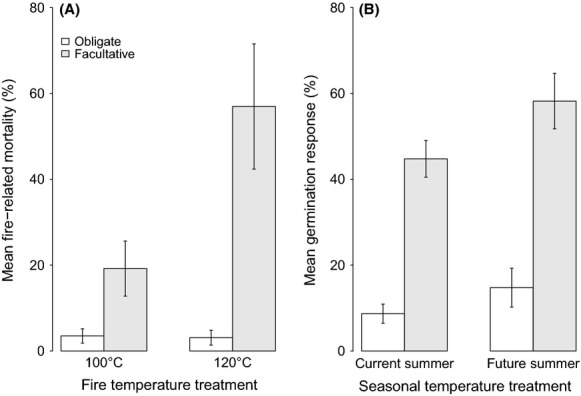
Comparisons between the obligate and facultative pyrogenic dormancy release groups for (A) mean fire-related mortality at 100°C and 120°C and (B) mean germination response to current and future summer-related temperatures.

Facultative species displayed much higher mortality than obligate species in response to typical fire-related soil temperatures (Fig. 6A). Increased summer gap temperatures related to climate change had a much greater impact on the facultative group than on the obligate group of species, by increasing levels of germination (Table 3) (Fig. 6B). Differences in the timing of germination (Table 2) were small but significant between the two groups for *T*_on_ (*W* = 790.5, *P *=* *0.028) with facultative species taking longer to germinate than the obligate group. A similar though nonsignificant difference was also found at T_50_, indicating that the obligate group may have some capacity to place seedlings more quickly into the postfire environment.

## Discussion

There is currently a lack of understanding of the full range of seed attributes and seed bank responses that maximize population persistence in fire-prone environments. We identified a suite of key seed traits and attributes and found that response to fire-related temperatures was the best indicator for assigning species to putative groups. Germination responses of six of the 14 co-occurring species studied were tightly bound to temperatures that only occur during fires. We classified these six species as members of a group with obligate pyrogenic dormancy release and found that associated seed bank responses for this group included high initial dormancy levels, resilience to decline in response to summer soil temperatures, and evidence of a postfire bet-hedging capability. The remaining study species, while still able to respond to fire-related temperature cues, had lower temperature thresholds for breaking dormancy and committed at least 30% of seeds to germinate after treatments mimicking summer gap temperatures that occur during the interfire period. These species were classified as having facultative pyrogenic dormancy release. A strong positive relationship between germination levels after low-temperature fire-related treatments and summer gap treatments suggests that this group will display the greatest response to temperatures generated during summer or in gaps. Lower limits of temperature thresholds required to break dormancy have received little research attention (Santana et al. 2013), but are likely to be important in determining potential (or residual) gap-recruiting ability and the level of response under inherently variable fire intensity (Ooi et al. 2012).

The six species assigned to the obligate pyrogenic dormancy class produced a flush of germination only when treatment temperatures of between 80°C and 100°C were reached; less than 20% germination occurred at temperatures below this. Similar thresholds have been found for species in other regions (Herranz et al. 1998; Ferrandis et al. 1999; Williams et al. 2004), suggesting that it is heat generated from fire, rather than other factors, that is the main driver. Soil temperatures this high are recorded during the passage of moderate-to-high severity fires (Bradstock and Auld 1995; Penman and Towerton 2008). Soil temperatures produced in gaps in heath or woodland vegetation in the absence of fire do not reach these levels, with a mean of 40°C during dry summer days and a peak of 55°C recorded in summer in the study region (Ooi et al. 2012), similar to those found in other studies or in other fire-prone habitats (Raison et al. 1986; Auld and Bradstock 1996; Baeza and Roy 2008; Santana et al. 2013).

For the eight facultative species, there was a large flush of germination at the lowest fire-related temperatures. These lower dormancy thresholds can therefore also promote germination at temperatures produced in the soil during the interfire period, germinating to between about 20% and 50% in response to summer gap temperature treatments. While able to persist in a fire-prone environment, these species do not have dormancy cues as tightly bound to fire. Several lines of evidence, in addition to their summer gap germination response, support the fact that seed traits of the facultative group of species are less tightly bound to fire than their obligate counterparts. At 100°C treatments, a typical soil temperature during high severity fire at depths of 1 to 3 cm (e.g., Shea et al. 1979; Auld 1986; Moreno and Oechel 1991; Bradstock and Auld 1995), seed mortality of facultative species was higher than that of the obligate species. Bet-hedging capacity was also very low for the facultative group, with a mean residual proportion of dormant seeds of 2.0% compared to 21.0% for the obligate species. Many of the facultative species displayed greater than 90% (and often 100%) dormancy broken at low fire-related temperatures of 60°C. Soil temperatures between 40°C and 60°C have been recorded during low severity fires or at greater depths during higher severity fires (recorded up to 7 cm depth; Bradstock and Auld 1995; also Auld 1986; Raison et al. 1986; Penman and Towerton 2008). Committing all seeds to germinate provides no bet-hedging capability and doing this at temperatures related to low severity fires, where recruitment success is lower (Purdie 1977; Knox and Clarke 2006), would appear to be a less secure path to successful population recovery after fire. A low-temperature dormancy threshold would also mean that seeds would have dormancy broken at great depths during high severity fires and hence cause germination of seeds at depths from which effective germination may not be possible. This would reduce recruitment success for most species, with the chance of successful recruitment decreasing with decreasing seed size (Bond et al. 1999).

Why these two groups of species maintain such wide variation in threshold temperatures is open to question. Several authors have suggested that differences in dormancy thresholds between species contribute to maintaining species coexistence because of natural variation in fire attributes (Trabaud and Oustric 1989; Moreno and Oechel 1991; Tárrega et al. 1992; Herranz et al. 1998). Soil heating can be extremely variable, both spatially and temporally (e.g., Penman and Towerton 2008), which could explain how species from both of our groups currently persist. How one group of species developed dormancy thresholds obligated to fire, while the other did not is less clear. One hypothesis is that minimum threshold temperatures that break dormancy may change over time with burial. This is quite a simple mechanism that is yet to be tested, because most studies of threshold temperatures are conducted only on fresh seeds.

Because dormancy of the facultative group of species identified is less tightly bound to fire than the obligate species, each group is subsequently susceptible to different types of threat. For the obligate group, high-temperature thresholds mean that they are dependent on high severity fires. A series of low severity fires, such as an implemented hazard reduction fire regime, would exclude recruitment from this group, making them vulnerable to such management actions. For the facultative group, the low-temperature thresholds mean that they can lose seeds to failed germination events between fires, in response to summer gap soil temperatures. Classifying species into these groups can therefore enable better prediction of species persistence under different scenarios, with the facultative group at higher risk to projected climatic changes. The strong relationship between low fire-related temperature thresholds (e.g., 60°C) and response to future summer-related temperature treatments provides support for the feasibility of assigning functional groups based on the level of response of physically dormant seeds to low-range experimental fire treatments.

The facultative group of species are all common and widespread, which suggests that despite being less tightly bound to fire, losses from the seed bank between fires are within limits that still allow population persistence under current climate conditions. However, this residual summer-or gap-related response appears to leave this group of species more exposed to increased seed bank losses under future climate scenarios. Facultative species displayed increased dormancy losses of between 7% and 49% above current levels under future summer gap soil temperatures. This compares to < 2% response in four of the six species in the obligate group and an approximate 15% increase in dormancy losses for the remaining two, which occurred only at the extreme 30-day future heat wave treatment. Large increases in dormancy loss between fires would have a negative impact on population persistence by depleting the seed bank, thus reducing the number of seeds available to respond to the next fire event. The greater the reduction of the seed bank between fires, the more likely that there will be a significant negative impact on subsequent recruitment success and hence population persistence.

The scale of the impact will also depend on whether or not dormancy temperature thresholds can change under a warmer climate and how quickly these changes could take place. However, there is currently little understanding of the plasticity of dormancy temperature thresholds of native species, or how much the parental environment can influence subsequent thresholds of progeny. Some evidence shows that minimum dormancy threshold temperatures are related to parental environment (Ooi et al. 2012). Additionally, in a study investigating seed bank longevity in habitats where fire has been actively excluded for many decades and disturbance implemented via clearing, dormancy loss for two of four *Acacia* species was very high over a three-year period (Orscheg and Enright 2011), suggesting perhaps that there has been selection for summer (nonpyrogenic) recruitment dormancy mechanisms. For now, a key challenge for assessing the level of impact that climate change will have in fire-prone regions is to gain an understanding of maternal effects and plasticity of seed dormancy thresholds.

Dormancy that is broken by temperatures that can only occur during fire provides strong evidence that these dormancy thresholds are a fire adaptation (Keeley et al. 2011; Moreira and Pausas 2012). Similar conclusions regarding fire adaptations have been made about some serotinous species, where seed release from cones in the canopy seed bank cannot occur until the resin seal has melted during burning (He et al. 2011; Midgley and Bond 2011). However, it is not clear whether the dormancy thresholds within our facultative group are indicative of a residual seasonal adaptation or are fire-adapted but have the negative effects of their lower dormancy thresholds offset by other traits. For example, as mentioned earlier, low dormancy thresholds could reduce bet-hedging ability by exhausting the seed bank. While serotinous species generally commit all seeds to the one fire event (Keeley et al. 2011), this strategy is often offset by seeds much larger in size than soil seed bank species (e.g., those from the genus *Banksia* or postfire flowering species such as *Telopea speciosissima*; Denham et al. 2011) or some limited temporal bet-hedging such as by a slow release of seeds via wet/dry cycles resulting from rainfall (Cowling and Lamont 1985; Whelan and Brown 1998). There was some support that facultative species in our study spread germination over a slightly longer time period than the obligate species, which could be indicative of some bet-hedging capability. To make strong conclusions regarding dormancy thresholds and offsets, further work is required, using data for a much larger number of species than we have used in this study. From the evidence to date, however, it appears that low-temperature thresholds maintained by these species are associated with higher fire-related mortality and reduced bet-hedging ability.
